# Chinese tree shrew: a permissive model for in vitro and in vivo replication of human adenovirus species B

**DOI:** 10.1080/22221751.2021.1895679

**Published:** 2021-03-13

**Authors:** Xiao Li, Zhichao Zhou, Wenkuan Liu, Ye Fan, Yinzhu Luo, Kangtian Li, Zhenxia Zheng, Xingui Tian, Rong Zhou

**Affiliations:** a State Key Laboratory of Respiratory Disease, National Clinical Research Center for Respiratory Disease, Guangzhou Institute of Respiratory Health, the First Affiliated Hospital of Guangzhou Medical University, Guangzhou Medical University, Guangzhou, People’s Republic of China; b Guangdong Provincial Key Laboratory of Laboratory Animals, Guangdong Laboratory Animals Monitoring Institute, Guangzhou, People’s Republic of China

**Keywords:** Tree shrew, human adenovirus, permissive animal model, pneumonia, vaccine

## Abstract

Human adenovirus (HAdV) species B can cause severe acute respiratory diseases. However, the researches to combat this infection have been hampered by the lack of an animal model permissive to the virus. Here, we report *in vitro* and *in vivo* HAdV species B infections of tree shrews, the closest relative of primates. HAdV-3, -7, -14, and -55 efficiently replicated in primary cell cultures. After intranasal inoculation of tree shrews with HAdV-55, the viral replication in the oropharyngeal region remained high until day 5 post-infection and was still detected until day 12. HAdV-55 in the lung or turbinate bone tissues reached the highest levels between days 3 and 5 post-infection, which indicated viral replication in the upper and lower respiratory tracts. HAdV-55 infection caused severe interstitial pneumonia in the animal. IL-8, IL-10, IL-17A, and IFN-γ expression in the peripheral blood mononuclear cells from infected animals was up-regulated. The pre-vaccination with HAdV-55 cleared the virus faster in the respiratory tract, mitigated lung pathological changes. Finally, HAdV-55 infection was propagated among tree shrews. Our study demonstrated that the tree shrew is a permissive animal model for HAdV species B infection and may serve as a valuable platform for testing multiple anti-viral treatments.

## Introduction

Human adenoviruses (HAdVs) are non-enveloped, double-stranded DNA viruses of the family Adenoviridae [1]. To date, seven species A–G and over 100 types of HAdVs have been identified (http://hadvwg.gmu.edu/), according to genome sequencing [2]. Among the different types of HAdV, the highly contagious ones cause many diseases, such as acute respiratory disease (ARD), gastroenteritis, cystitis, and keratoconjunctivitis [3].

HAdVs species B include the types HAdV-3, -7, -14, -55, and -21, which have been associated with severe ARD outbreaks and have caused severe and even fatal infections in both children and adults [4-8]. HAdV-3 and -7 are the most common types that affect pediatric patients with ARD [9-11]. HAdV-7 and -21 are the prevalent types that cause febrile ARD outbreaks in the military [10,12-16]. Since 2006, two re-emerging types, HAdV-14 and -55, have caused numerous outbreaks among both civilian and military populations [17-19]. HAdV-55, a highly virulent pathogen, has emerged in northern China causing severe and fatal pneumonia [9,20-22]. HAdV-55 is an inter-typic recombinant of HAdV-11 and HAdV-14, identified as type 11a in early studies [23-25]. HAdV-11 species B is the predominant type observed in patients with haemorrhagic cystitis [26]. Until now, no commercial vaccine or anti-viral drug that prevents or cures HAdV infection is available, although some drugs and vaccine candidates are under development [12,27,28].

Animal models of adenovirus infection are critical for studying pathogenicity, vaccine and medicine development, and preclinical evaluation of oncolytic adenoviral vectors. However, a permissive animal model, which can support HAdV species B infection and replication, is lacking, possibly due to a strict host range restriction. Initially, the replication of HAdV was restricted to human cells. However, some animal models have been established for HAdV-5 species C infection, including cotton rat, hamster, New Zealand rabbit, pig, and non-human primate [29,30]. The HAdV species A, C, D, E, F, and G use the coxsackie and adenovirus receptor (CAR) as their primary cellular attachment receptor [31]; whereas, HAdV species B infect cells through the receptor desmoglein 2 (DSG2) or CD46 [32]. Although HAdVs can infect rodent cells through CAR, they cannot effectively replicate in these cells due to host range restriction factors (HRRF) [33,34]. Further, small rodent animals, such as cotton rat and Syrian hamster, are still the most often used models for infection with HAdV-5 and oncolytic adenoviruses [35]. Conversely, HAdV species B cannot infect or effectively replicate in rodent cells due to unknown HRRF [36]. Therefore, the aim of this study was to identify a permissive model, susceptible to HAdV-B infection both *in vitro* and *in vivo*.

*Tupaia belangeri*, colloquially known as tree shrew, which is a small mammal currently placed in the order Scandentia, is the closest relative of primates [37,38]. A whole-genome phylogenetic analysis revealed a genetic relationship between Chinese tree shrews and human beings [39]. Based on their physiological properties, tree shrews have been established as viable animal models alternative to rodents and primates for investigations of nervous, behavioural, mental, metabolic, and immune-related diseases, cancer, and especially infectious diseases [40]. Currently, tree shrews have been used as animal models for hepatitis virus B, C, D, and E, herpes simplex viruses type 1 and 2 [40,41].

To establish an animal model for HAdV species B, we infected primary epithelial cells from mouse, golden hamster, cotton rat, New Zealand rabbit, small pig, and tree shrew and determined that rAd3EGFP infected primary cells from Chinese tree shrews and effectively and productively replicated. Furthermore, tree shrew cells were also susceptible to HAdV-7, -14 and -55 infections and replications. More importantly, HAdV-55 replication reached maximum levels in the lung or turbinate bones at 3–5 days post-infection (d.p.i) after *in vivo* intranasal administration of HAdV-55 to tree shrew. Finally, we established a tree shrew model to evaluate vaccines against HAdV-55 infection. Altogether, our study established the first permissive animal model for HAdV-B infection and replication, which can be used in virological studies.

## Materials & methods

### Virus strains and cells

HAdV-7 GZ08 (GenBank no. GQ478341.1), HAdV-14p1 GZ01 (GenBank no. JQ824845.1), and HAdV-55 Shanxi-Y16 (GenBank no. KF911353.1) strains were maintained in our laboratory [42], and the competent HAdV-3-based vector rAd3ΔE3GFP (GZ1strain, DQ099432.4) was generated by our team [43]. All viruses were cultured in HEp-2 cells or AD293 cells obtained from the ATCC and subsequently maintained in our lab [42]; HAdV particles were purified by standard caesium chloride gradient centrifugation and suspended in phosphate-buffered saline (PBS) (pH 7.4), as previously described [44].

### Animal ethics statement

All institutional and national guidelines for the care and use of laboratory animals were followed. This study was carried out in strict accordance with the guidelines of Guangdong Regulation for Administration of Laboratory Animals (2010), and the guidelines on the welfare of non-human primates. All animal infection experiments were performed under animal biosafety level 2 conditions in the Guangdong Laboratory Animals Monitoring Institute (GDLAMI) (Guangzhou, China), which is accredited by the Association for Assessment and Accreditation of Laboratory Animal Care (AAALAC) International. The animal protocol was approved by the Institutional Animal Care and Use Committee (IACUC) of GDLAMI (Ethics number: I-IACUC2015002). Six- to eight-month-old male tree shrews, weighing 105–150 g, were obtained from the Animal Experimental Centre of Kunming Medical University (Kunming, China), which were approved by Kunming Department of Science and Technology [approval ID, SCXK (DIAN) K2013-0002] and kept healthy and well-nourished with strict feeding protocols and close monitoring of their health status prior to and during the entire study period. Tree shrews were individually housed in cages, specifically developed for their behaviours, and then sedated and humanely euthanized with sodium pentobarbital solution administered by a licensed veterinarian at the GDLAMI.

### HAdV growth characteristics in primary tree shrew cells

Two healthy tree shrews were sedated and humanely euthanized to collect their lung, trachea, and kidney tissues. Then the primary cells from the fresh tissue samples were isolated and cultured as described previously [30]. The primary cell monolayers in 24-well plates were infected with 100 TCID_50_ of HAdV-55, -7, -14, or rAd3-EGFP, for 1 h at 37°C. Then the monolayers were washed twice with MEM and incubated for 7 d. The infected cells were then observed daily under microscopy to check the cytopathic effect (CPE) or green fluorescent signals.

To detect viral replication, the infected cells and the culture medium were harvested at 24, 48, 96, and 144 h post-infection (h.p.i). The viral genomic DNA was extracted with a TaKaRa MiniBEST Viral RNA/DNA Extraction Kit Ver. 5.0 (TaKaRa, Dalian, China) according to the manufacturer’s instructions, and the viral genomic DNA copies were determined by quantitative PCR (qPCR), as described below.

To check whether the primary cells produced infectious progeny viruses after adenovirus infection, cells were harvested at 48 or 72 h.p.i and subjected to three freeze–thaw cycles and centrifuged at 10,000 × g for 30 min to remove cell debris. The virus supernatant was serially diluted to infect HEp-2 and the CPE was analyzed between 2 and 7 d by microscopy to calculate TCID_50_ according to the Spearman & Kärber algorithm method [45].

### Taqman qPCR

TaqMan real-time PCR was performed to quantify the number of copies of the conserved hexon region presented in most HAdV types. The primer and probe sequences were:

Hexon probe, 5′-(FAM)AAAACAACGGAGCAGCCA(BHQ1)-3′

Hexon forward primer, 5′-AAGGCGGTCAGGCAAAACC-3′

Hexon reverse primer, 5′-CCATGTCAATATCATATTCGACTTTCTGA-3′

The primers and the probe were synthesized by TaKaRa (China). The premix ex amplification was conducted using 10 pmol of primers, 3 pmol of probe, and 5 *μ*L of DNA in a final volume of 25 *μ*L. Cycling conditions included an initial incubation at 94°C for 2 min, followed by 40 cycles of 94°C for 10 s and 55°C for 35 s. qPCR was performed with the Applied Biosystems 7500 Real-Time PCR System (Life Technologies, Singapore) using our optimized reaction components and cycling conditions.

### Virus microneutralization (MN) assays

The MN assays were performed as described previously [42]. Sera heated at 56°C for 30 min were serially diluted (2-fold) in DMEM, and 50 *μ*L aliquots of each dilution were mixed with 50 *μ*L of HAdV-55 (50% tissue culture infective doses [TCID_50_] = 100). The antibody-virus mixtures were incubated at 37°C for 1 h and transferred to 96-well plates containing 70–90% confluent monolayers of AD293 cells. The monolayers were observed under the microscope at 96 h. The neutralization titres were determined according to the highest serum dilution that completely inhibited any visually observable CPE.

### Animal experiments

Male tree shrews were housed in separate cages and allowed to acclimatize for one week prior to vaccination or infection. Pre-existing neutralizing antibody (NAb) levels of tree shrews against HAdV-55 were detected by the MN assay. The tree shrews with pre-existing NAbs against HAdV-55 (>32) were excluded from further experiments.

For this investigation, three animal studies were designed. The first one characterized the tree shrew model by intranasal infection with HAdV-55. Briefly, nineteen male tree shrews were randomly divided into two groups, treatment (*n* = 15) and control (*n* = 4) groups. Animals were lightly anaesthetized with 1% isoflurane before inoculation. Each animal in the treatment group was intranasally infected with 100 *μ*L of 5 × 10^5^ TCID_50_ HAdV-55; meanwhile, those in the control group were inoculated with an equal volume of PBS (PH 7.4). All tree shrews were monitored daily for clinical signs, body weight, and body temperature. Oropharyngeal swab samples from each tree shrew were also collected daily for viral analysis. Before measuring their body temperatures, the animals were kept quiet for 10 min. On 1, 3, 5, and 7 d.p.i, three tree shrews from the treatment group and one from the control group were euthanized, and the lung and turbinate bone tissues were collected for viral titration. The remaining tree shrews from the treatment group were monitored daily for 7 more days for body weight change and oropharyngeal swab sample collection. They were humanely euthanized on 14 d.p.i. and their lung and turbinate bone tissues were collected for viral titration. HAdV genomic DNA in each animal tissue sample was extracted using QIAamp DNA Mini Kit (Qiagen Co. Ltd., Shanghai, China) according to the manufacturer’s protocols. To check the antibody responses after intranasal infection, the sera from HAdV-55-infected tree shrews were collected on 1, 3, 5, 7, and 14 d.p.i and the NAb concentrations were determined by ELISA or SVN.

The second animal study established a tree shrew model for HAdV-55 vaccine evaluation. Briefly, 23 male tree shrews were randomly assigned into three groups: A (MOCK) (*n* = 4), B (immunized) (*n* = 11), and C (control) (*n* = 8) (Table 1). After acclimatization, tree shrews in groups A and C were intramuscularly vaccinated with 100 µL of MEM (mock), and tree shrews in group B were intramuscularly vaccinated with 100 µL of 2 × 10^10^ of inactivated HAdV-55 VPs (inactivated with 20 mmol/L beta-propiolactone at 4°C for 24 h)[46]. On days 14 and 28, two booster vaccinations identical to the first were given to the same tree shrews. Ten days after the final vaccination (day 38), aliquots of 20 *µ*L sera from the tree shrews were collected for NAb detection. On day 39, the tree shrews were intranasally challenged with 100 *μ*L of 5 × 10^5^ TCID_50_ of HAdV-55 (group B and C) or 100 *μ*L of MEM. On day 3 post-challenge, 6 tree shrews from group B and 4 tree shrews from group C were humanely euthanized and their lungs, turbinate bones, and PBMC were collected. The other animals were euthanized on day 5 post-challenge and their tissues were collected.

**Table 1.** Assignment of tree shrew for immunization and virus challenge.

Group (n)	Vaccination / Boost	Challenge
A (MOCK) (4)	MEM	MEM
B (immunized) (11)	Inactivated HAdV-55	HAdV-55
C (control) (8)	MEM	HAdV-55

The third animal trial investigated whether HAdV-55 could be transmitted among tree shrews. Six tree shrews were randomly divided into 3 groups (*n* = 2 per group), and the animals for the same group were placed in one cage. One animal from each group was intranasally infected with 2 × 10^5^ TCID_50_ of HAdV-55, and the other group was not infected. All tree shrews were monitored for clinical signs and body weight daily, and their oropharyngeal swab samples were collected for virus analysis daily.

### Indirect ELISA

Mouse monoclonal antibodies against tree shrew IgG-Fc were prepared with a standard hybridoma method and labelled with horseradish-peroxidase (HRP) [47] by Guangzhou Ruida Bio Co. Ltd. (M0160-HRP) to be used as secondary antibodies. Antigen-specific IgG responses in tree shrews were then detected by ELISA. Briefly, 96-well Nunc MaxiSorp™ flat-bottom plates (Nunc, Roskilde, Denmark) were coated with purified heat-inactivated HAdV-55 (10^10^ VPs/mL) (inactivated at 56°C for 30 min) using a carbonate buffer (pH 9.6) and incubated overnight at 4°C. Wells were then washed once with PBS and the unspecific binding of the antibodies was blocked by incubating the wells with 2% bovine serum albumin in PBST (PBS + 0.1% Tween 20) for 2 h. The plates were washed and incubated with serially diluted tree shrew serum solutions in 1% BSA/PBST. After incubation for 1 h at 37°C, the plates were washed four times with PBST and incubated with a 1:5000 dilution of the previously described HRP-conjugated mouse anti-tree shrew IgG-Fc, M0160-HRP, in 1% BSA/PBST for 1 h at room temperature. The plates were washed with PBST and the colorimetric reaction was initiated with the addition of TMB substrate (Thermo Scientific, Rockford, IL, USA). The reaction was stopped with 50 *μ*L 2 M H_2_SO_4_, and the optical density (OD) was measured at 450 nm on an ELISA plate reader (Multiskan MK3; Thermo Scientific, USA).

### Histopathology

Tissues were fixed with 10% neutral buffered formalin and embedded in paraffin for haematoxylin and eosin (HE) and immunohistochemical staining. The tissue sections were treated according to standard procedures [48] and blindly assessed by a board-certified pathologist. For immunohistochemical staining, anti-HAdV-55 mouse serum was used as the primary antibody to detect HAdV-55 antigens, and anti-PBS mouse serum was used as negative control.

### Quantitative reverse transcription-PCR (qRT-PCR)

The qRT-PCR was performed to detect the expression of cytokines: interleukin (IL)-6, IL-8, IL-10, IL-17A, and interferon-γ (IFN-γ), and of the housekeeping gene glyceraldehyde-phosphate dehydrogenase (GAPDH) in tree shrews. Tree shrew PBMC were extracted using a mouse PBMC separation kit (TBD, Tianjin, China), and kept in 500 *μ*L TRIzolTM Reagent (Invitrogen, Shanghai, China) at −80°C until total RNA extraction was performed. The total number of RNA copies was extracted according to the manufacturer’s protocol. To determine the mRNA levels of various cytokines, which expression were induced by HAdV-55 infection, one-step real-time qRT-PCR was performed with total RNA and with the Applied Biosystems 7500 Real-Time PCR System (Life Technologies, Singapore), using our optimized reaction components and cycling conditions. Each 25 *μ*L reaction mixture contained 1× reaction buffer (50 mM Tris-Cl [pH 8.9], 75 mM KCl, 4 mM MgCl_2_, 10% glycerol), 0.6 mM deoxynucleoside triphosphates (dNTPs) (Promega, Beijing, China), 0.4 *μ*M primer (BGI, Shenzhen, China), 0.12 *μ*M fluorescent probe (TaKaRa, China), 50 U of M-MLV reverse transcriptase (Promega, Beijing, China), 1 U of Taq DNA polymerase (Promega, China), and 5 *μ*L of template RNA. The cycling conditions were 48°C for 10 min, 94°C for 2 min, and then 40 cycles of 94°C for 10 s and 55°C for 35 s. Cytokine mRNA levels were normalized to those of the housekeeping gene *GAPDH*. All sequences of qPCR primers and probes are listed in Table S1.

### Statistical analysis

Statistical analysis was performed using GraphPad Prism 5.0 software. Differences among multiple groups were analyzed by a two-way ANOVA with Tukey’s multiple comparisons test. The data are expressed as the mean ± standard deviation (SD). The body temperature changes after infection were analyzed by one-way ANOVA with Dunnett's Multiple Comparison Test. The differences in parameters between the treatment and control groups were analyzed by one-tailed unpaired t test. A probability (P) value of <0.05 was considered statistically signiﬁcant. Significant differences between groups are denoted by *(*P* < 0.05), **(*P* < 0.01), ***(*P* < 0.001), or not significant (ns) (*P* > 0.05).

## Results

### Tree shrew primary cells supported in vitro replication of HAdV species B

To screen for laboratory animals permissive to HAdV species B infection, a replication-competent, E3-deleted HAdV-3-based vector encoding GFP (rAd3ΔE3GFP) was used to infect a panel of primary cells from kidney and/or lung of different animals, which included small pig, mouse, golden hamster, Wuzhishan minipig, and tree shrew. Only primary cells from Wuzhishan minipig and tree shrew were effectively infected by rAd3ΔE3GFP and visualized by a GFP signal (data not shown). However, the levels of infectious virus progeny produced in the minipig cells were very low, indicating that these cells were semi-permissive to HAdV-3 infection, which coincides with the results of a previous report [49].

**Figure 1.**
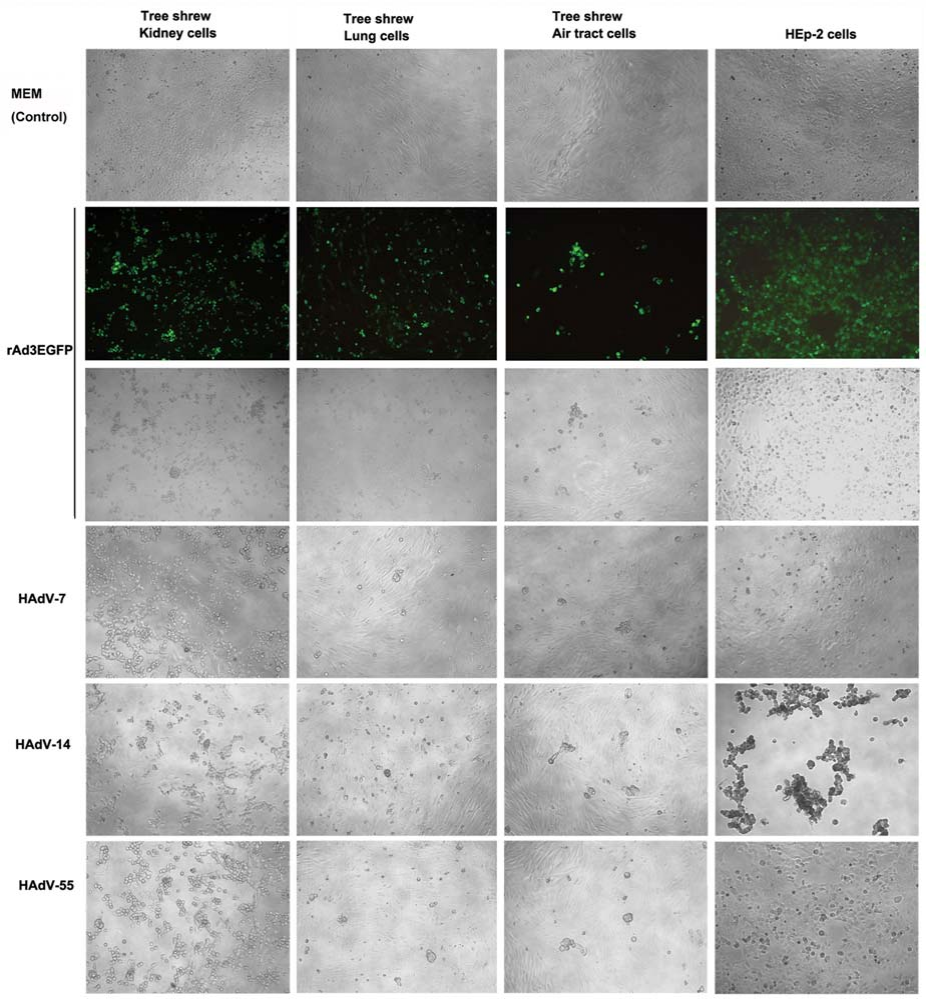
Tree shrew primary cells infected by HAdV species B. The primary cells from kidney, lung, and trachea of tree shrew were infected with 100 TCID_50_ of HAdV-55, -7, -14, or rAd3-EGFP. Human laryngeal carcinoma cell line HEp-2 was infected by the HAdVs as the control. Cells were observed at 72 h post-infection. Cells infected with rAd3-EGFP were also observed under a fluorescence microscope (200×).

Contrary to the study on minipig cells, HAdV-55, -7, -14, or rAd3-EGFP infected primary cells from kidney, lung, and trachea of tree shrew with 100 TCID_50_. Infection of the permissive human HEp-2 cells was a positive control. As shown in Figure 1, at 48 h.p.i., all four HAdVs caused nearly complete CPE on tree shrew kidney cells, and the GFP fluorescence was observed in cells infected with rAd3-EGFP. In contrast, the infection of tree shrew lung and trachea cells was less severe, but CPE was visualized. No difference in the infectivity of these viruses was noted.

**Figure 2.**
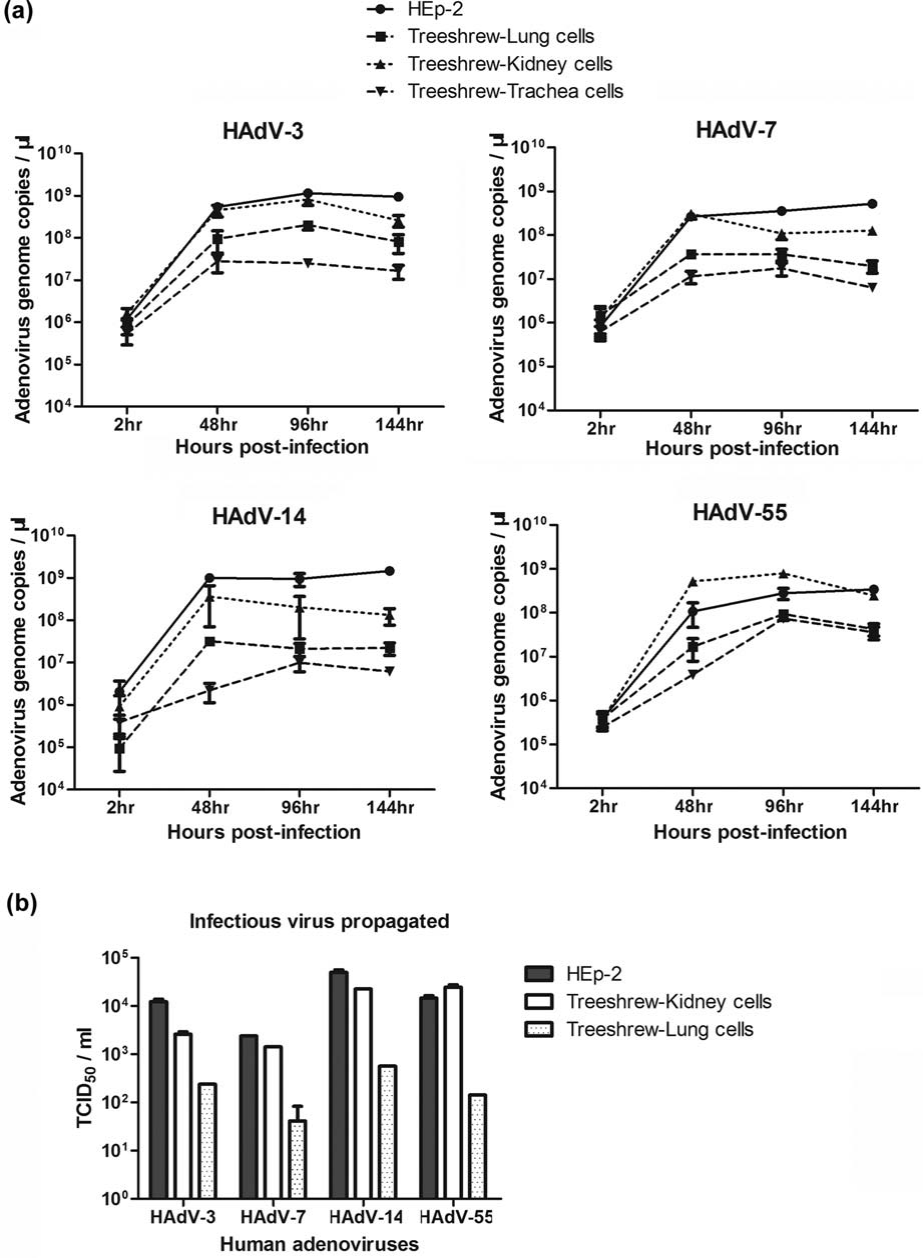
Primary tree shrew cells supported the effective replication of HAdV-55, -7, -14, or -3. (a) HAdV-55, -7, -14 or -3 genomic DNA copies were detected in primary tree shrew cells of kidney, lung, and trachea on 2, 48, 96, and 144 h post-infection by qPCR. (b) The amount of infectious HAdV-3, -55, -7, or -14 progeny produced from the tree shrew cells at 48 h post-infection were measured by infecting HEp-2 cells. HEp-2 cells were infected in parallel with HAdV-55, -7, -14, or -3 as the control.

To determine the replication efficiency of HAdV-3, -7, -14, and -55, samples of primary cultures of different tree shrew cells infected with these viruses were collected at various time points for viral nucleic acid detection (Figure 2(a)). To compare the viral growth between tree shrew and human cells, HEp-2 cells were infected in parallel. All four viruses replicated well on human HEp-2 cells at 48 h.p.i.; the replication reached the highest level at 96 h.p.i., which was maintained up to 144 h.p.i. The replication efficiency of all HAdV viruses in kidney cells was the highest, and comparable to that in HEp-2 cells. Although the viral replication in tree shrew trachea and lung cells was not as high as that in human HEp-2 and tree shrew kidney cells, the replication time courses were the same.

The infectivity of HAdV-3, -55, -7, and -14 progenies produced from various tree shrew cells was further tested by incubating them with HEp-2 cells (Figure 2(b)). Surprisingly, at 48 h.p.i., the levels of infectious HAdV-3, -55, -7, or -14 progenies from primary tree shrew kidney cells were similar to those from highly permissive human HEp-2 cells. However, less but detectable progeny viruses from primary tree shrew lung cells were produced. Wild-type HAdV-55 and rAd3-EGFP, propagated for ten generations in tree shrew primary kidney cells, showed no decline in infectious viral titres.

### HAdV-55 infected and replicated in the respiratory tract of tree shrew

Based on the results of the *in vitro* study, we further tested the *in vivo* replication and pathogenicity of HAdV species B. Before performing the experiments, the sera of 18 tree shrews were analyzed for the presence of pre-existing neutralizing antibodies (NAbs) against HAdV-3, -7, -14, and -55 (Table 2). Six and one samples were positive for NAbs against HAdV-3 and HAdV-55, respectively (NAb titre >32). No NAbs were detected against HAdV-7 or HAdV-14. All tree shrews with pre-existing NAbs against HAdV-55 (NAb titre >32) were excluded from further vaccination and infection.

**Table 2.** Positive rate of neutralizing antibodies against different human adenovirus types in tree shrew (*n* = 18).

Human adenovirus	Positive samples ^a^	Negative samples	Positive rate (%)
HAdV-3	6	12	33.33
HAdV-7	0	18	0.00
HAdV-14	1	17	5.56
HAdV-55	0	18	0.00

^a^ Sera sample with neutralizing antibody titre >32 was defined as positive.

**Figure 3.**
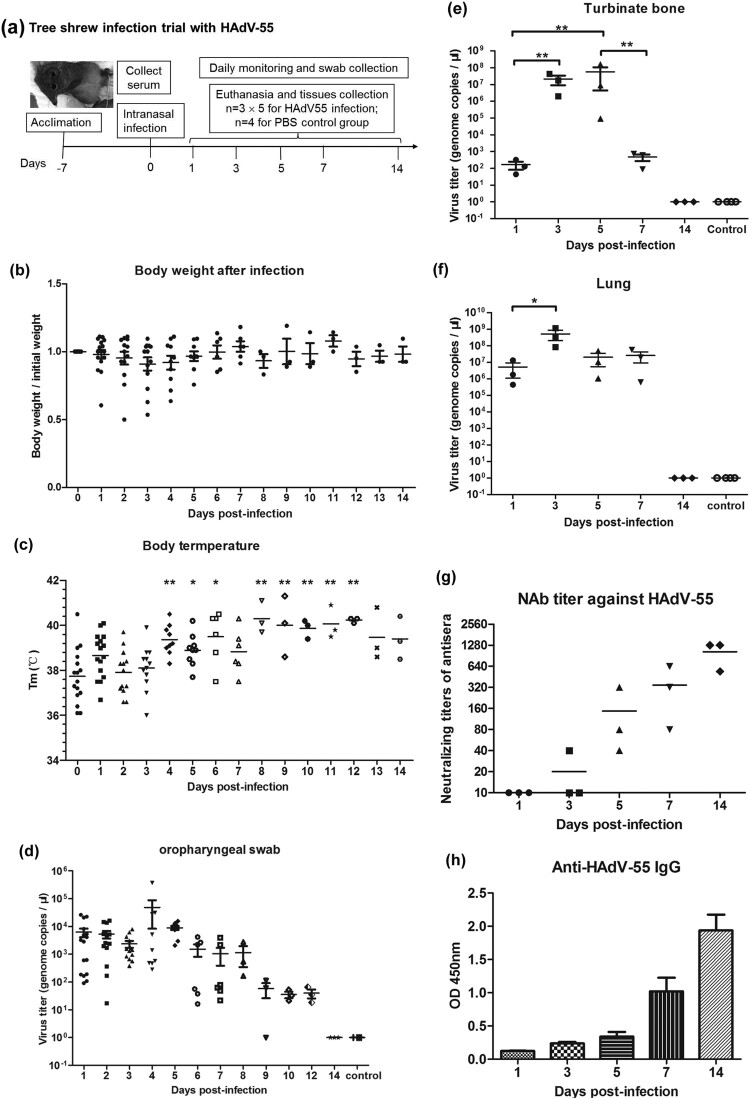
Body weight and body temperature change and viral genomic DNA detection in HAdV-55-infected tree shrews. (a) Experimental scheme of the tree shrew infection with HAdV-55. Fifteen tree shrews were intranasally inoculated with 5 × 10^5^ TCID_50_ of HAdV-55. Four control tree shrews were treated with PBS. All tree shrews were daily monitored for behaviour, body weight, and body temperature. Oropharyngeal swabs from each tree shrew were collected daily for viral load monitoring. At 1, 3, 5, 7, and 14 d post-infection (d.p.i), three tree shrews from the treatment group were euthanized and lung and turbinate bone tissues were collected for viral titration. One tree shrew from the control group was necropsied at 1, 3, 5, and 7 d.p.i. HAdV genomic DNA in animal tissue samples was extracted using QIAamp DNA Mini Kit (Qiagen Co. Ltd., Shanghai, China), followed by qPCR to quantify the viral DNA copies. (b) Body weight changes of tree shrews after infection with HAdV-55. (c) Changes of rectal temperature of tree shrews infected with HAdV-55. Statistically significant differences are denoted by * (*P* < 0.05), ** (*P* < 0.01); versus 0 d.p.i. (d) Viral DNA load in oropharyngeal swabs. (e) Viral DNA load in turbinate bone. (f) Viral DNA load in lung tissues. Each symbol represents an individual tree shrew and the horizontal lines indicate the mean values or the mean ± standard deviation (SD). (g) The neutralizing antibody titres against HAdV-55 in sera from HAdV-55-infected tree shrews were detected by a micro-neutralization test. (h) HAdV-55-specific IgG antibodies in sera from HAdV-55-infected tree shrews were detected by ELISA.

Tree shrews were intranasally inoculated with 5 × 10^5^ TCID_50_ of HAdV-55 or PBS (Figure 3(a)). Body weight and temperature were monitored for 14 d. All shrews survived the duration of the trial. Several tree shrews infected with HAdV-55 exhibited rapid weight loss greater than 20% of their initial body weight within 5 d.p.i (Figure 3(b)). Uninfected tree shrews had an average body temperature of 37.7°C, ranging from 36.1°C to 40.5°C. Tree shrews infected with HAdV-55 exhibited significant increase in temperature at 1, 4, 5 and 6 d.p.i., but not on day 2 and 3 post-infection (Figure 3(c)).

To investigate the HAdV-55 replication efficiency in the respiratory tract, tree shrew oropharyngeal swap samples were collected daily (Figure 3(d)); meanwhile, turbinate (Figure 3(e)) and lung tissues (Figure 3(f)) were collected on days 1, 3, 5, 7, and 14 post-infection. No HAdV-55 genomic DNA was detected in all tree shrew samples of mock infection with PBS. From 1 to 3 d.p.i., the HAdV-55 DNA levels in oropharyngeal regions were between 102–104 genome copies/μL, and continuously increased on 4–5 d.p.i. Subsequently, the viral DNA levels decreased about 10 times less on average from the peak, remaining at the plateau in the next 3 d. From 9 d.p.i. until later, the viral DNA levels continuously declined and reached undetectable levels by day 14 (Figure 3(d)), whereas the HAdV DNA was still detected at 12 d.p.i. in all swab samples.

The viral titre of HAdV-55 in turbinate bones at 3 d.p.i was about 10^7^ genome copies/μL, which was 10^5^-fold higher than that of 1 d.p.i (Figure 3(e)). The viral titre continuously increased up to 5 d.p.i. and then dropped on 7 d.p.i.

Interestingly, the viral titre in the animal lung quickly increased on 1 d.p.i., almost reaching 10^7^ genome copies/μL, and peaked on 3 d.p.i, the latter increased over 100-fold compared to that on 1 d.p.i (Figure 3(f)). On 5 and 7 d.p.i., the lung viral titres still maintained at high levels; and by 14 d.p.i., no HAdV-55 DNA was detected in the tree shrew samples. Antibody responses against HAdV-55 were detected in intranasally infected tree shrews (Figure 3(g and h)) with some variability among animals. On 5 d.p.i., the NAbs of one infected tree shrew reached a high titre of 320; meanwhile, the other two infected animals had detectable NAbs titres of 40 and 80 (Figure 3(g)). On 14 d.p.i., all of them exhibited higher NAb titres (640–1280). BALB/c control mice, which were intranasally infected with the same HAdV-55 dose, generated NAbs at much lower levels on 14 d.p.i., ranging from undetectable levels to a titre of 40 (data not shown). HAdV-55-specific IgG NAbs were detected on 3 d.p.i at a low level by ELISA (Figure 3(h)) and increased to higher levels until 14 d.p.i.

**Figure 4.**
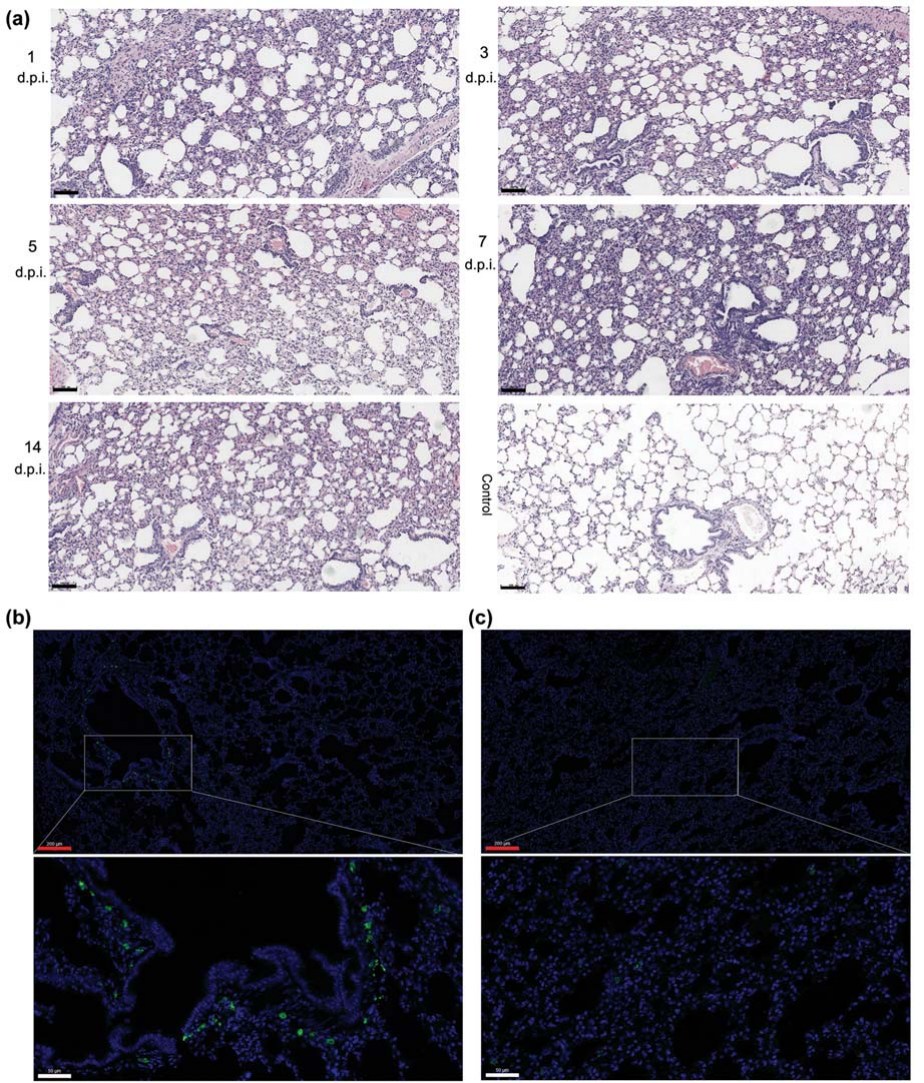
Histopathological lung changes in tree shrews infected with HAdV-55 at 1, 3, 5, 7, and 14 d post-infection. (a) Haematoxylin and eosin (HE) staining of the lung tissues. (b) Immunofluorescence analysis of HAdV-55 in the lung tissues of HAdV-55-infected tree shrew at 3 d post-infection. (c) Immunofluorescence analysis of HAdV-55 in lung tissue of control tree shrew treated with PBS. A mouse polyclonal antibody raised against HAdV-55 virions was used for HAdV-55-specific staining. Black scale bar, 100 μm; red scale bar, 200 μm; white scale bar, 50 μm.

The pulmonary pathology of tree shrews infected with HAdV-55 on 1, 3, 5, 7, and 14 d.p.i was examined histopathologically (Figure 4). All infected tree shrews had mild or moderate interstitial widening from 1 to 14 d.p.i (Figure 4(a)). No obvious inflammatory exudate in alveolar spaces was observed. Multifocal lymphocytic infiltration was noted on 1 and 7 d.p.i, and a small amount of lymphocyte infiltration was detected on 3 and 5 d.p.i. Surprisingly, the arteriolar wall was markedly thickened, and the lumen was narrowed on 1 and 3 d.p.i. Bronchial epithelial hyperplasia was detected on 3 d.p.i., and obvious alveolar epithelial bronchioles were found on 5 and 7 d.p.i. Especially on 7 d.p.i., the compensatory dilatation of partial alveolar cavity was observed. On 14 d.p.i., no obvious pathological change was detected in the lung tissues, except for mild alveolar epithelial bronchioles and mild interstitial thickening. Immunofluorescent microscopic analysis using HAdV-55-specific antibodies indicated the presence of HAdV-55 antigens in the lungs of HAdV-55-infected tree shrews (Figure 4(b)), as a difference compared to the control animals (Figure 4(b)). These results suggested that HAdV-55 infected the respiratory tracts of tree shrews, resulting in interstitial pneumonia.

### Pre-vaccination inhibited in vivo HAdV-55 replication

**Figure 5.**
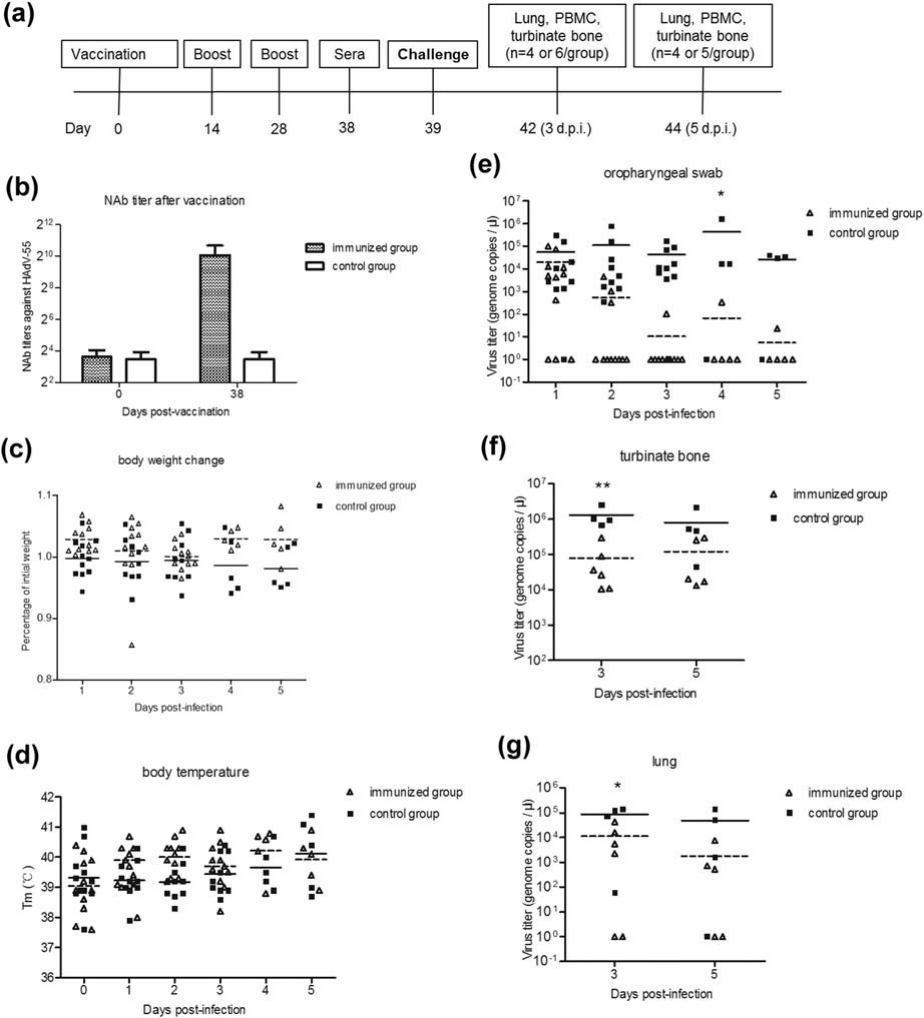
HAdV-55 challenge on pre-vaccinated tree shrews. (a) Experimental scheme of tree shrew challenge studies. Eleven tree shrews (immunized group) were intramuscularly vaccinated with inactivated HAdV-55 virions and 8 (control group) were intramuscularly vaccinated with MEM. Sera from the tree shrews were collected for NAb detection. The tree shrews were challenged with HAdV-55, and then humanely euthanized on days 3 and 5 post-challenge to collect their blood and tissues. Body weight, body temperature, and behaviour were measured daily. Viral loads in tissues and daily swabs were monitored. (b) The neutralizing antibody titres against HAdV-55 in sera from vaccinated tree shrews were detected by a micro-neutralization test. (c) Body weight and (d) body temperature was monitored at the indicated time points. (e) Viral DNA load in oropharyngeal swabs. (f) Viral DNA load in turbinate bone. (g) Viral DNA load in lung tissues. Each symbol represents an individual tree shrew, and the horizontal lines indicate the mean values or the mean ± standard deviation (SD). Statistically significant differences between the immunized and control groups are denoted by * (*P* < 0.05), ** (*P* < 0.01).

To establish a tree shrew model for vaccine evaluation, the experimental groups were intramuscularly vaccinated with HAdV-55 and then intranasally challenged with HAdV-55 (Figure 5(a)). After three vaccinations, all immunized tree shrews generated NAbs at titres of 256–2048. The control group (injected with MEM) did not show detectable NAbs (<32) (Figure 5(b)). After vaccination, the tree shrews were intranasally challenged with HAdV-55. No significant differences in body weight and temperature between the infection-alone group and the immunization/challenge group were noted throughout the study (Figure 5(c and d)).

The HAdV-55 viral titres from the oropharyngeal regions of the tree shrews remained high in all the animals from the infection-alone group at 5 d.p.i., except for one sample, in which HAdV-55 was undetectable at 1, 4, and 5 d.p.i. (Figure 5(e)). In contrast, out of 11 swab samples from the immunization/challenge group, eight had no detectable HAdV-55 virus at 2 d.p.i., three showed a relatively low viral titre, and only one was positive at 3, 4, and 5 d.p.i. A significant difference in HAdV-55 viral titres between both groups at 3, 4 or 5 d.p.i. was noted (*p* < 0.05).

At 3 and 5 d.p.i. tree shrews were sacrificed, and their lung and turbinate bone tissues were collected. The HAdV-55 titres in turbinate bones or lung tissues of the immunization/challenge group were significantly lower than those in the corresponding tissues of the control infection-alone group at 3 d.p.i. (*p*<0.05) (Figure 5(f and g)). No obvious pathology was observed in the lungs of tree shrews from the MOCK group (Figure S1a). Three days after infection, HAdV-55 caused severe interstitial pneumonia in the infected tree shrews in the control infection-alone group (Figure S1c). Specifically, severe thickened alveolar walls were observed due to congestion and infiltration of a large number of monocytes and lymphocytes into the alveoli. In contrast, HAdV-55 infection led to moderate interstitial pneumonia in the tree shrews that were in the pre-immunized/challenge group (Figure S1b). Altogether, these results indicated that the pre-vaccination of tree shrew generated effective immune responses, which could provide a partial protection to the animal against homologous infection.

To further understand the pathogenesis and immunological responses, we assessed the cytokine gene expression in the peripheral blood mononuclear cells (PBMC) of the tree shrews infected with HAdV-55 by qRT-PCR (Figure S2). IFN-γ, a type-II interferon which has anti-viral, immunoregulatory, and anti-tumour properties, is produced by activated lymphocytes after exposure to specific antigens. IL-6 and IL-17A are two pro-inflammatory cytokines produced by activated T cells, IL-8 is a major inflammatory mediator, and IL-10 is an anti-inflammatory cytokine that inhibits other cytokine syntheses. We first established the real-time qRT-PCR method to detect the transcription levels of these cytokine genes. The specificity, sensitivity, and linear correlation (coefficient R^2^ ≥ 0.99) of our method were good in a wide linear range of mRNA copy numbers, from 10 copies (IL-6, IL-17A), 100 copies (IL-10, GAPDH), up to 1,000 copies (IL-8, IFN-γ). The lowest detectable values of IL-6, IL-8, IL-10, IL-17A, IFN-γ, and GAPDH were 8, 8, 4, 8, 128, and 4 copies, respectively. Compared with the non-treated animals (MOCK group), the transcription of IL-10 and IFN-γ in the PBMC of the animals which only received HAdV-55 challenge and mock vaccination with MEM (control group) was significantly up-regulated at 3 d.p.i. (*p* < 0.05) (Figure S2). Meanwhile, the IL-8 (*p* < 0.05), IL-17A (*p* < 0.01), IL-10 (*p* < 0.001), and IFN-γ (*p* < 0.01) expression increased on 5 d.p.i., compared to that of the MOCK group. On 3 d.p.i., IL-10, IL-17A, and IFN-γ mRNA levels of the PBMC from the immunized group were significantly lower than those from the control group. In contrast, IL-8 mRNA levels from the immunized group were significantly higher than those of the control group. Interestingly, IL-6 mRNA levels in the immunized group slightly fell at 5 d.p.i. compared to those at 3 d.p.i.; however, IL-6 mRNA levels in the control group slightly increased at 5 d.p.i.

### HAdV-55 transmission among tree shrews

**Figure 6.**
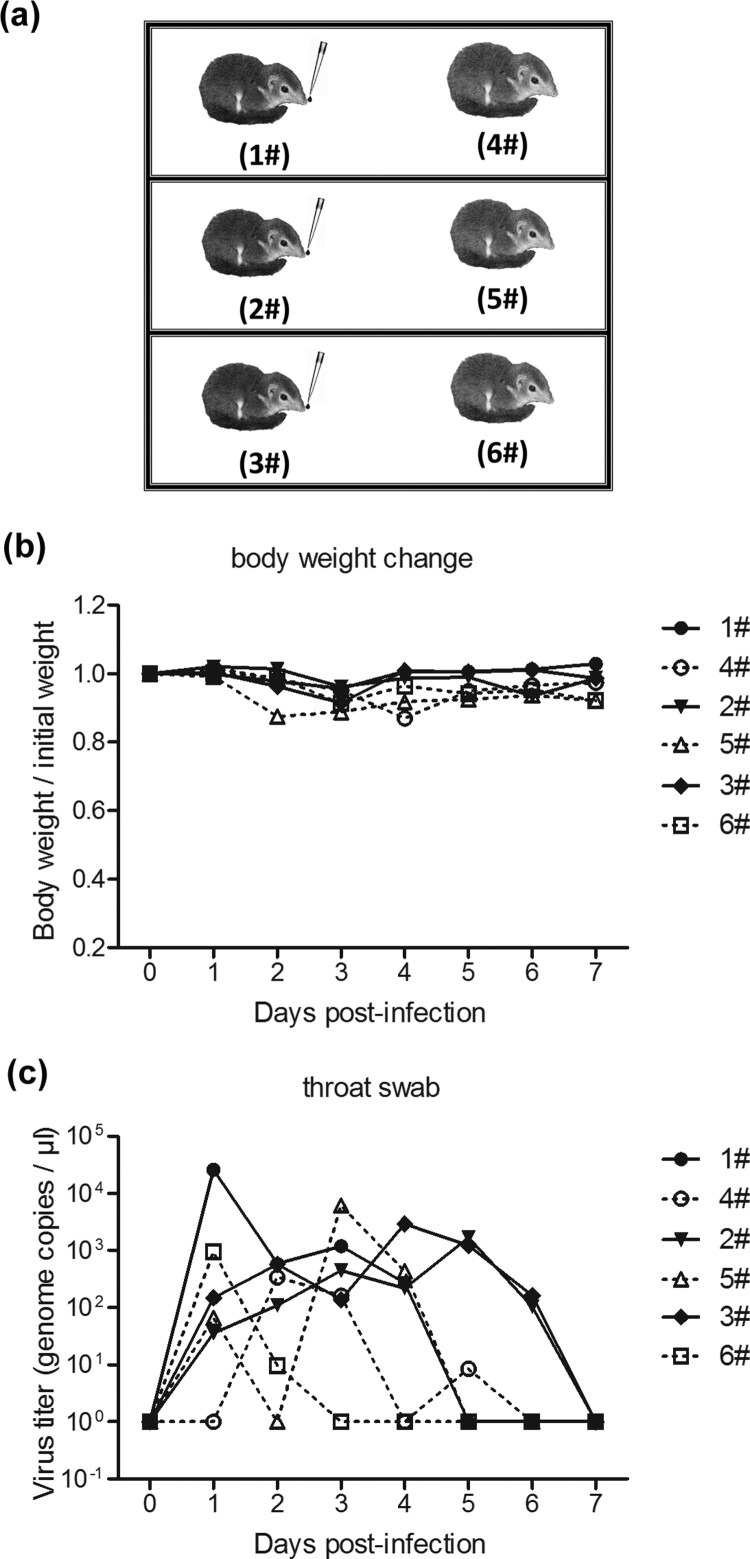
HAdV-55 experimental transmission among tree shrews. (a) Six tree shrews were separated into three independent cages and one animal from each cage was inoculated with HAdV-55. The tree shrews were monitored daily for body weight (b); oropharyngeal swab samples from each tree shrew were collected daily for viral DNA levels by qPCR (c).

Three pairs of tree shrews were housed in three independent cages. One animal from each cage was intranasally inoculated with HAdV-55 (1#, 2#, and 3# tree shrews; infected group), the other three tree shrews (4#, 5#, and 6#; uninfected group) were not infected (Figure 6(a)). All tree shrews exhibited about 4–10% body weight loss on 3 d.p.i. (Figure 6(b)). Tree shrew 1# exhibited relatively high titres of HAdV-55 in oropharyngeal swab samples at 1–4 d.p.i. but it was not detected at 5 d.p.i or later (Figure 6(c)). Tree shrews 2# and 3# presented relatively high titres of HAdV-55 in oropharyngeal swab samples at 1–6 d.p.i. but it was not detected at 7 d.p.i. Interestingly, HAdV-55 genomic DNA was detected in respiratory samples of naïve tree shrews, 4#, 5#, and 6# (Figure 6(c)). A relatively high titre of HAdV-55 genomic DNA was detected in the oropharyngeal swab samples of 4# tree shrew at 2 and 3 d.p.i., of 5# at 1, 3, and 4 d.p.i., and of 6# at 1 and 2 d.p.i. This result suggested that HAdV-55 might spread among tree shrews via either direct contact or droplets.

## Discussion

Adenoviruses have a restricted host range for infecting epithelial cells from monkeys, dogs, and rodents, resulting in a much lower or no virus progeny than when human cells are infected [30]. Jogler et al. demonstrated that the HAdV-5 vector replicated *in vitro* on porcine cells to high titres [30]. However, Griesche et al. indicated that porcine cells were semi-permissive to species B [49], and receptor human DSG2 humanized mouse cells did not support HAdV-3 full replication [36]. To screen for animals permissive to HAdV species B infection, a panel of primary cells from mouse, golden hamster, minipig, dog, and Chinese tree shrew were cultured. We confirmed that primary porcine kidney and lung cells were semi-permissive to HAdV-3, -14, -55 and -7; meanwhile, primary mouse, golden hamster, and dog cells were not infected by these viruses (data not shown).

Only primary tree shrew cells were susceptible to HAdV-3, -14, -55, and -7 infection and produced high titres of infectious virus progeny and CPE, similar to human cells (Figure 1 and 2). These results demonstrated that tree shrew supported *in vitro* HAdV species B infection and replication. Tree shrew cells were also permissive for HAdV-4 (species E) and HAdV-5 (species C) (data not shown), suggesting that tree shrew cells may get infected by most HAdV types, which will be investigated in our future studies.

The increasing viral titres in turbinate bone, lung tissues, and oropharyngeal swab samples demonstrated that tree shrew supported *in vivo* HAdV-55 intranasal infection and replication (Figure 3). Viral titres kept high levels till 8 d.p.i. in the early oropharyngeal swab samples, and the viral load was highest at 4 d.p.i. at 4.83 × 10^7^ copies/mL. Huh et al. reported prolonged viral shedding in immunocompetent adults with HAdV-55 respiratory infection, with the highest viral load in the earliest respiratory samples at 8.69 log_10_ copies/mL [50].

Moreover, it was interesting to discover that some tree shrews had low titres of pre-existing NAbs against HAdV-3, indicating that tree shrew may be naturally infected by HAdV-3 (Table 2). We also noted that HAdV-55 was efficiently transmitted from inoculated to naïve tree shrews by direct contact or via aerosols (Figure 6). Although this tree shrew model maybe imperfect and there are several questions related to this model, such as whether the virus transmitted from inoculated tree shrew caused pathogenesis in lung, bronchia or other tissues of naïve tree shrew, or whether this virus could be transmitted to other tree shrews, which will be answered in our future work. Recently, Feng Y et al. reported that although both human desmoglein-2 and human CD46 mediate HAdV-55 infection, but human desmoglein-2 plays a major role [51]. Amino acid sequence alignment showed 81.24% similarity between desmoglein-2 of Homo sapiens and Chinese tree shrew (Tupaia chinensis), which is higher than that between Homo sapiens and Sus scrofa (78.41%), and Mus musculus (76.18%), but lower than between Homo sapiens and Gorilla gorilla gorilla (97.5%), and Macaca mulatta (96.26%). The CD46 similarity between Homo sapiens and Chinese tree shrew was only 47.73%, which is still higher than that between Homo sapiens and Sus scrofa (43.39%), and Mus musculus (46.68%), but lower than that between Homo sapiens and Gorilla gorilla gorilla (98.47%), and Macaca mulatta (82.91%). Thus, HAdV55 may recognize tree shrew desmoglein-2 but not CD46 for infection; however, a series of experiments needs to be performed to draw a conclusion. However, all these results demonstrated that tree shrew is a useful *in vitro* and *in vivo* HAdV-B replication animal model.

Tree shrews might also be a relevant animal model to study the pathogenesis of HAdV-induced infection. The clinical symptoms and signs in children or adults infected with HAdV-55 were fever, cough, rhinorrhoea, swelling of tonsils, and pneumonia [8,50,52]. We observed lung injury after HAdV-55 infection, and many tree shrews suffered from a snotty nose similar to rhinorrhoea in human patients. Although no other obvious clinical signs were observed in most animals, a few tree shrews experienced weight loss and elevated body temperature, possibly due to individual variability. More importantly, HAdV-55 caused interstitial pneumonia in all infected tree shrews, which is in line with infection reported in human cases [8,52].

In addition, tree shrews had increased IL-6, IFN-γ, IL-10, IL-17, and IL-8 levels in the acute period of HAdV-55 infection (Figure S2). Lymphocytic inflammation occurs as a response to virus infection by the innate and adaptive immune systems. Tissue injury happened in some cases due to the release of pro-inflammatory cytokines. Chen et al. studied the relationship between peripheral blood cell profiles and the severity of HAdV-55 infection in humans [53]. They found that patients with different disease severities due to HAdV-55 infection had significantly different immune responses. Patients with severe infections had significantly higher numbers of IL-17+CD4+ cells and higher levels of IFN-γ, IL-10, and IFN-α2. Earlier studies also noted that patients with adenoviral respiratory infection had strong inflammatory responses and higher serum IL-6 levels than with influenza and RSV infection [54,55]. Lim et al. found that the IL-6, IL-10, and IFN-γ levels were significantly associated with hypoxemia in patients with HAdV-55 pneumonia [56]. This study only analyzed the expression of cytokine genes in PBMC. Thus, further research on cytokine levels in infected organs, such as the lung and liver, might provide more information about the pathogenesis of HAdV infection. This study suggested that tree shrew might be a useful model for the study of HAdV pathogenesis.

After HAdV-55 intranasal infection, most tree shrews rapidly produced high titres of antibody responses. As early as 3 d.p.i., low levels of IgG were observed in two tree shrews (Figure 3(g and h)). Furthermore, the NAbs generated by pre-vaccination prevented infection by HAdV-55 and provided partial protection (Figure 5), which suggested that humoral immunity could play a role in fighting adenovirus infection, although cellular immunity might also be responsible for the protection, which should be further investigated. Although the pre-existing immune response generated by intramuscular injection of HAdV-55 enhanced the virus clearance in swab, turbinate bone, and lung samples (Figure 5), HAdV-55 was still detected and caused moderate lung pathology in some tree shrews of the immunized group at 3 or 5 d.p.i. (Figure 5). The reason for the partial protection is unclear and will be further studied. Thus, tree shrew could be a valuable model for evaluating HAdV-B vaccines.

The tree shrew (*Tupaia belangeri*) is a small laboratory animal that has gained increasing attention in China and worldwide. The whole-genome of the Chinese tree shrew was sequenced and analyzed in 2013, showing that the immune, nervous, and metabolic systems were very close to those of humans [39]. Additionally, the tree shrew offers many advantages as a model for the study of human diseases, such as small size, easy feeding, short reproductive and life cycles, and high reproductivity [57]. However, there are still several hurdles to overcome. First, tree shrew strains with pure genetic background are still being established. So far, scientists in Kunming of China have generated the fourth offspring by inbreeding [40]. In this study, we used the first or second offspring for infection. Additionally, some reagents for working with tree shrew are missing, such as tree shrew-specific antibodies. In this study, we developed tree shrew IgG-specific secondary antibodies for ELISA and established a real-time qPCR method to measure the expression of several cytokines. However, tree shrew IgM- and IgG subtypes- and IgA-specific antibodies should be developed. Moreover, tree shrew innate immune system should be characterized and methods to measure cytokine levels should be established. Notably, the lack of permissive animal model is a limitation for developing oncolytic HAdV vectors. Chinese tree shrews may be used to establish an animal model for the preclinical evaluation of oncolytic HAdV vectors.

Adult tree shrews are easier to acquire and handle. Therefore, we first tried to infect adult tree shrews with HAdV-55. However, HAdV-3 and some other HAdV types are more likely to infect infants and children. Cao et al. found that patients who had HAdV-55 were about 10 years older and had higher pneumonia severity index scores than those with other types of HAdV (HAdV-7, HAdV-3, HAdV-14, HAdV-50, and HAdV-C), and the systemic blood pressure was also higher among patients in the HAdV-55 group [52]. In addition, earlier literature reported tree shrew adenovirus causing fatal infection in infant tree shrews [58]. In our future studies, new-born tree shrews will be infected with more types of HAdV to compare the pathogenesis in adult tree shrews.

In summary, we determined that the small laboratory animal, tree shrew, was susceptible to different types of HAdV-B infections and replication *in vitro* and *in vivo*, and we established a tree shrew model for HAdV-55 infection. The established model enables the study of HAdV-B pathogenesis, evaluation of vaccines and therapeutic drugs, and preclinical evaluation of oncolytic HAdV-B vectors. However, more detailed pathological changes and HAdV-55 distribution in the respiratory tract and other organs, as well as by other types of species B, need to be investigated in the tree shrew model.

## Supplementary Material

Fig_S2_cytokines.tif

Fig_S1_HE_smaller.tif

supplementary_materials.docx
