# Supplementary figures and images for: Chinese tree shrew: a permissive model for in vitro and in vivo replication of human adenovirus species B
Source: Emerg Microbes Infect. 2021 Mar 13;10(1):424–38. doi: 10.1080/22221751.2021.1895679 (PMC7971223; doi:10.1080/22221751.2021.1895679)

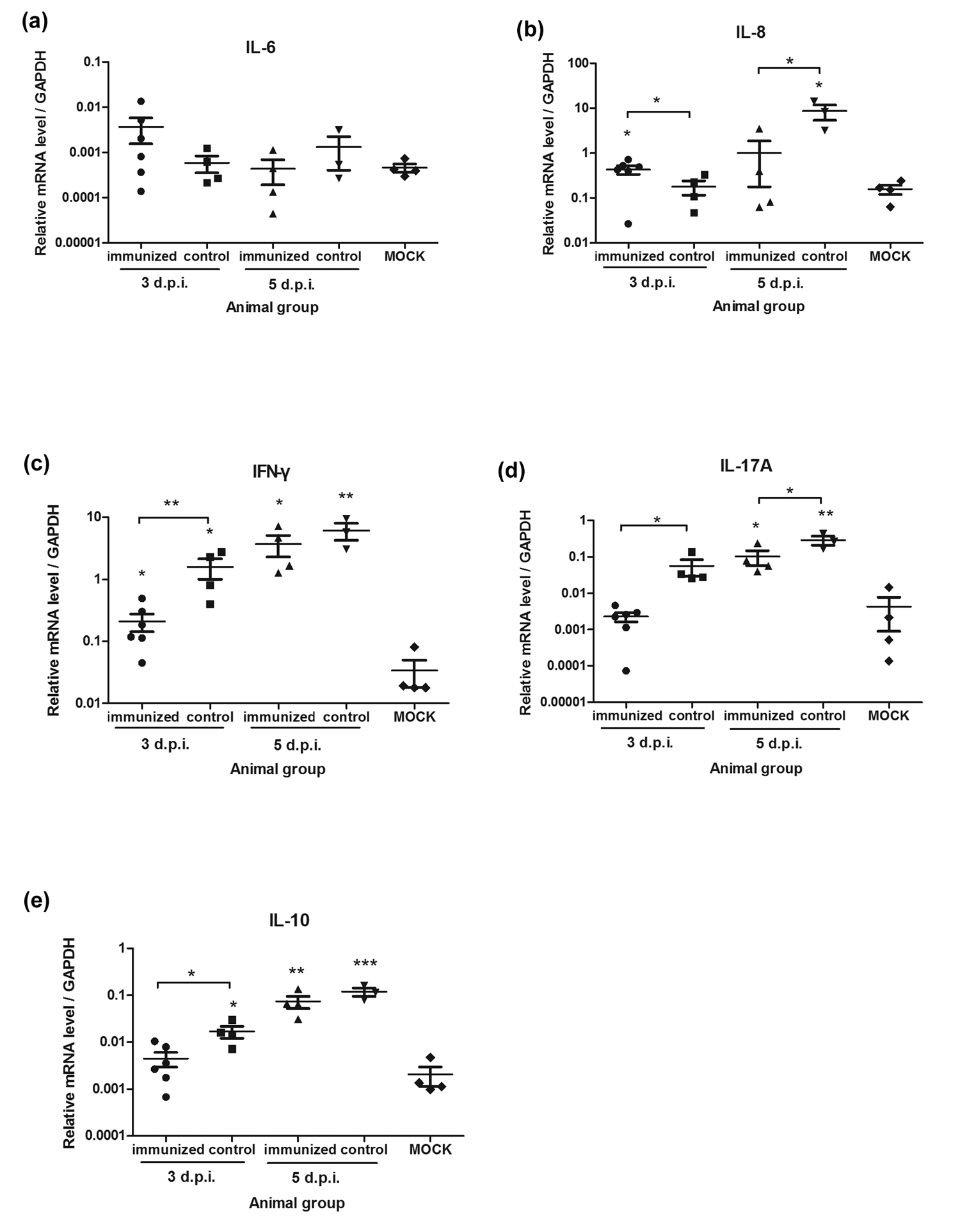

Supplement: Fig_S2_cytokines.tif [file TEMI_A_1895679_SM6299.tif]

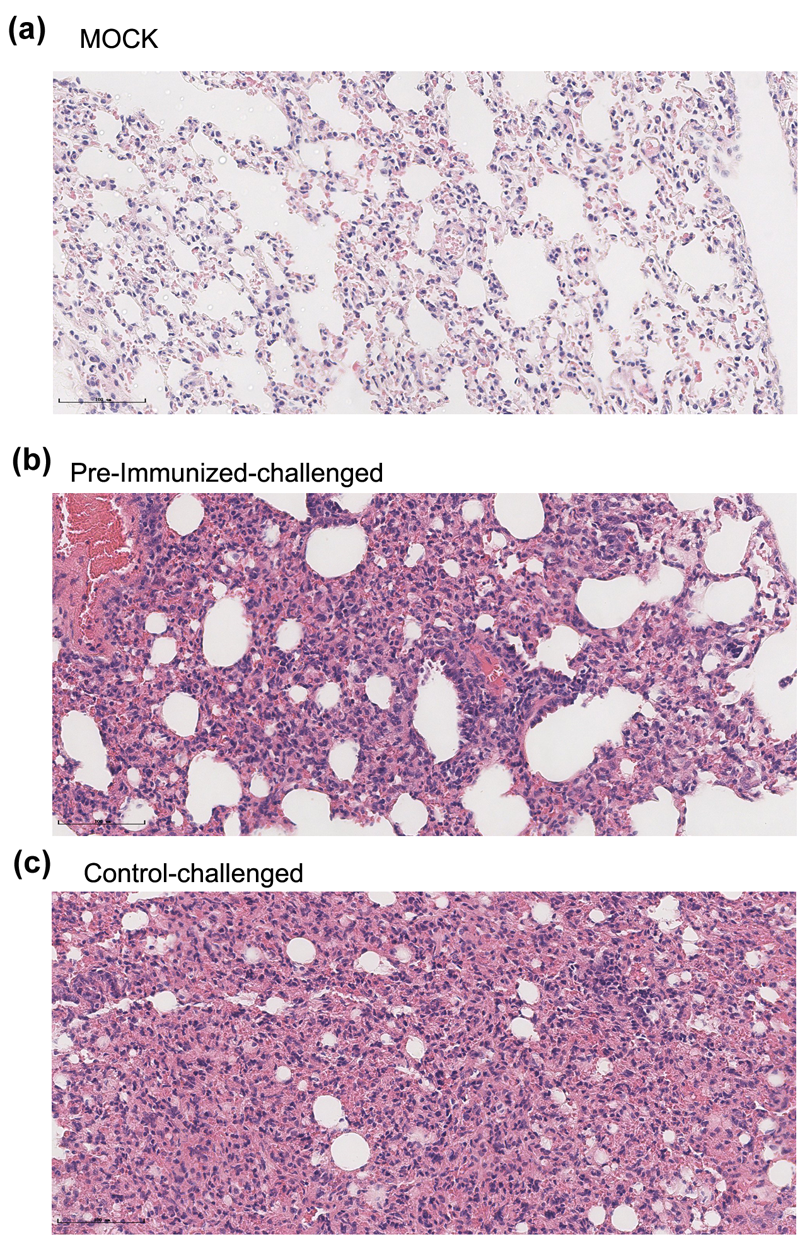

Supplement: Fig_S1_HE_smaller.tif [file TEMI_A_1895679_SM6298.tif]
