# Supplementary material for: Chinese tree shrew: a permissive model for in vitro and in vivo replication of human adenovirus species B
Source: Emerg Microbes Infect. 2021 Mar 13;10(1):424–38. doi: 10.1080/22221751.2021.1895679 (PMC7971223; doi:10.1080/22221751.2021.1895679)
Supplement: supplementary_materials.docx [file TEMI_A_1895679_SM6297.docx]

**Supplementary materials**

**Figure legends**

**Figure S1. Histopathology of the lungs of HAdV-55-challenged tree shrews.** Lung samples were fixed, sectioned, and stained with hematoxylin and eosin (H&E). (A) Tissues from the mock group. (B) Tissues from HAdV-55-challenged tree shrews in the pre-immunized group. (C) Tissues from HAdV-55-challenged tree shrews in the control group. Tissues show moderate interstitial pneumonia and thickening of the alveolar walls (B), or severe interstitial pneumonia and severe thickening of the alveolar walls (C). Scale bars represent 100 μm.

**Figure S2. Cytokine gene transcription levels in the PBMC of** **HAdV-55-challenged tree shrews.** Cytokine mRNA levels of IL-6, IL-8, IL-10, IL-17A, and IFN-γ in tree shrews PBMC were measured by qRT-PCR. Samples were harvested on the indicated post-infection times. Tree shrews in MOCK group were immunized with DMEM and then challenged with DMEM. Each sample was tested in triplicate. Each symbol represents an individual tree shrew and the values were presented as the means ± SD. Statistically significant differences between the immunized and control groups are denoted by * (*P* < 0.05), ** (*P* < 0.01).

**Tables**

**Table S1. Primers and probes used in real-time RT-PCR studies**

| **Target** | **Primer and probe** | **Sequence (5′-3**′**)** |
| --- | --- | --- |
| **IL-6** | T-IL-6F | 5′- TGCTGAAGCAAAAGGAAACGT -3′ |
|  | T-IL-6R | 5′- CCATCAAGCTGGCATTTGAA -3′ |
|  | T-IL-6Probe | 5′- FAM-CCAAGTATAGTTCCCACCCCTGACCCA-BHQ1 -3′ |
| **IL-8** | T-IL-8F | 5′- GACTTCCAAGCTGGCTATTGCT -3′ |
|  | T-IL-8R | 5′- TTCTTGTCAAAACTGCAGCTTCA -3′ |
|  | T-IL-8Probe | 5′- Texas red-TTGGCAGCCTTAATGTTCTCTGCAGCTC-BHQ2 -3′ |
| **IL-10** | T-IL-10F | 5′- GGGAGGGTGAAGACTTTCTTTCAA -3′ |
|  | T-IL-10R | 5′- CTTCAGCAGAGACTCACTCAGCAA -3′ |
|  | T-IL-10Probe | 5′- FAM-CCAGTGATCAGCTGGACA-BHQ1 -3′ |
| **IL-17A** | T-IL-17F | 5′- CAAAAGAGCCTCAGATTACTACAAACG -3′ |
|  | T-IL-17R | 5′- GGGATATCTCTCAGGGTCCTCAT -3′ |
|  | T-IL-17Probe | 5′-Texas red-TCCACTTCACCTTGGACTCTCCACCG-BHQ2 -3′ |
| **IFN-γ** | T-IFN-γF | 5′- TGCTTTCTATCATTTTGGGATCTTC -3′ |
|  | T-IFN-γR | 5′- TCCTGGATCAGTTGCATTAAAATAGT -3′ |
|  | T-IFN-γProbe | 5′- FAM-AGCTGTTACTGCCAGGCCCCATTTATG-BHQ1 -3′ |
| **GAPDH** | T-GAPDH-F | 5′- AAGGTCGGAGTAAACGGATTTG -3′ |
|  | T-GAPDH-R | 5′- CGTTGATGGCCACGACATC -3′ |
|  | T-GAPDH-Probe | 5′- JOE-TGGTCACCAGGGCTGCCTTCAAC-BHQ1 -3′ |
